# Bullying Victimization and Adolescent Depression, Anxiety and Stress: The Mediation of Cognitive Emotion Regulation

**DOI:** 10.3390/children10121897

**Published:** 2023-12-07

**Authors:** Mariacarolina Vacca, Silvia Cerolini, Anna Zegretti, Andrea Zagaria, Caterina Lombardo

**Affiliations:** Department of Psychology, Sapienza University of Rome, 00185 Rome, Italy; silvia.cerolini@uniroma1.it (S.C.); anna.zegretti@uniroma1.it (A.Z.); andrea.zagaria@uniroma1.it (A.Z.); caterina.lombardo@uniroma1.it (C.L.)

**Keywords:** bullying, cognitive emotion regulation, psychopathology, adolescents

## Abstract

Background: Existing research has revealed a robust association between bullying victimization and psychological distress, but less is known about the underlying mechanism of this link. cognitive emotion regulation (CER) strategies could be a potential mediator. The current study examined the role of functional and dysfunctional CER strategies as potential mediators of the association between bullying victimization and depression, anxiety, and stress symptoms among 638 high school students (53.9% boys; Mean age = 15.65, SD = 1.32). Method: Participants completed a series of questionnaires assessing bullying victimization (Olweus Bully/Victim Questionnaire), CER strategies (CERQ-18), and symptoms of depression, anxiety, and stress (DASS-21). The indirect relationships between bullying victimization and psychopathological symptoms via functional and dysfunctional CER strategies were tested through structural equation modeling. Results: Dysfunctional CER strategies mediated the impact of bullying victimization on depression, anxiety, and stress. In contrast, bullying victimization did not significantly influence functional CER strategies. Conclusions: The findings provide additional support for the detrimental role of bullying victimization on mental distress, also suggesting that this effect is not only direct, but indirect is well. These results are particularly relevant in light of the absence of mediation by protective factors such as the use of positive emotion regulation strategies.

## 1. Introduction

Bullying victimization is a social phenomenon consisting of repeated exposure to intentional negative actions from one or more individuals, accompanied by the perception of an interpersonal power imbalance between the perpetrator and the victim [1]. These aspects conceptually differentiate bullying from other forms of abuse [2,3], such as delinquency, sexual harassment, and physical aggression [4,5]. The oppressive actions exercised over victims can be distinguished in terms of direct or indirect forms of bullying. Direct bullying is easily noticeable because it includes explicit or face-to-face attacks on the victim expressed through physical (e.g., hitting, pushing, and tripping) or verbal aggressions (e.g., name-calling and insulting) [6]. In contrast, indirect or relational bullying is more unobtrusive and refers to secretive and insidious behaviors (e.g., gossiping, spreading rumors, and social exclusion; destroying one’s property) that intend to progressively isolate the victim from their peers through emotional maltreatment and by damaging their social status [7]. The experience of these forms of victimization could be particularly threatening during adolescence, a developmental period (10–19 years of age [8]) of multiple biological and psychological transitions culminating with the maturation of complex cognitive and behavioral abilities [8]. During adolescence, individuals enter an emerging social environment and need to establish new interpersonal relationships with peers [9,10]. During this period, the urge to establish dominant status [9], in response to the pronounced need for peer-group belonging and acceptance [10,11], increases; thus, experiencing discrimination and isolation can be exceptionally frustrating [12].

Prevalence studies in bullying have revealed that school is the most common site where intimidation occurs among adolescents globally [13,14,15] due to the different social class levels united in one place from morning to evening [15]. Data from the Global School-based Student Health Survey [16] suggested a global pooled prevalence of bullying victimization of 30.5% amongst adolescents, with rates varying according to students’ age, sex, socio-economic status, and peer/parental support perceived [15]. More specifically, it has been observed that, overall, being male, younger in age, having a below-average socioeconomic status, and receiving low peer and parental support were associated with a greater risk of bullying victimization [15]. Some authors have demonstrated that prevalence rates of bullying victimization in Europe are lower compared with those observed in Africa and America, although more recent evidence showed an overall noteworthy prevalence of 36.39% in European countries [17]. In the Italian school context, 20% of students between 11 and 17 years reported having been bullied two or more times in a month [18].

These alarming frequency estimates of bullying victimization are substantial, considering the consequences of bullying on adolescents’ development and adjustment, making this phenomenon a major public health challenge [19,20].

Negative acts from peers, when experienced over time, could be associated with developmental trajectories including emotional and behavioral difficulties [21]. Exposure to this form of interpersonal victimization can also undermine the brain’s functionality and connectivity [22], and thereby interfere with healthy development [21]. Concerning the effect on mental adjustment, a systematic review outlined that being victimized in youth was associated with mental distress and negative psychosocial outcomes, including increased peer rejection and poorer school performance and connectedness, both over the short (12 months) and long term (up to 8 years later) [23].

Recent meta-analytic evidence indicated significant associations between bullying victimization and psychological harm [24], sedentary behaviors [25], suicide attempts [26], lower academic achievement [27] peer rejection, and low school connectedness [23]. It is strongly evidenced that bullying victimization in adolescents is related to mental health difficulties, such as externalizing and internalizing symptoms [12,23,28,29]. Some authors, however, have underlined the usefulness of considering potential underlying mechanisms that may mediate this well-known association (e.g., sleep duration [30], resilience [31], and internet addiction [32]). One potential approach is employing cognitive emotion regulation (CER) strategies.

CER strategies consist of individual cognitive responses to emotion-eliciting events [33] and have been recognized as particularly relevant in the context of adolescent psychopathology [34]. The literature distinguishes functional and dysfunctional CER strategies by whether they can facilitate or impede individual functioning in coping with stressful events [35,36]. Functional CER includes strategies employed to process emotions, while the dysfunctional facet consists of strategies used to block or avoid negative emotions related to stressful events [34,36]. The development of CER strategies is crucial for adolescents considering that they encounter a variety of transitional challenges (pubertal development, emerging intimate relationships, and school changes), and need to develop cognitive abilities to effectively manage their emotions during these events [37,38,39]. In this perspective, emotional responses associated with bullying victimization adversely affect adolescents’ cognitive flexibility [40], and may thus negatively impact their CER [39]. Previous studies have reported a higher use of dysfunctional CER strategies (e.g., catastrophizing, self-blame, blaming others, and rumination) among bullied school students as compared with non-bullied school students [41], emphasizing the possibility that bullying victimization could be related to poor cognitive systems of emotion regulation [42]. Considering that a greater use of dysfunctional CER has been associated with high psychopathological symptoms in adolescents [43,44], CER strategies may be potential mediators of the association between bullying victimization and mental difficulties in this population. Indeed, previous studies have established emotion regulation as a key mediator in the maltreatment–psychopathology association [45]. More specifically, it has been suggested that considering the well-known association between experiences of maltreatment (e.g., emotional and physical) and emotion dysregulation in childhood, as well as between the latter and psychopathology, it is plausible that emotion dysregulation is a mediator in the maltreatment–psychopathology link [45].

In this respect, some findings on bullying victimization are available in the literature. For example, Gardner et al. [46] found that suppression and reappraisal positively mediated the relationship between high peer victimization and high loneliness in late childhood. However, they did not assess the effect on other psychopathological symptoms. In contrast, Labella et al. [47] found that specific emotion regulation strategies mediated the association of bullying victimization with depression. However, the authors did not evaluate cognitive strategies and used a sample of young adults. In view of the information presented above, the present study expanded previous research by evaluating the mediating role of functional and dysfunctional CER strategies in the relationship between bullying victimization and depression, anxiety, and stress in a sample of adolescents. More specifically, based on previous research, it was hypothesized that dysfunctional CER strategies would positively mediate this link, whereas functional CER strategies would act as negative mediators between bullying victimization and levels of psychopathological symptoms. Considering the effects of sex, age, and body mass index (BMI) on bullying victimization [26], CER strategies [48], and psychopathology [49], all these aspects were used as covariates in the tested mediation model.

## 2. Materials and Methods

### 2.1. Participants

**Figure 1:**
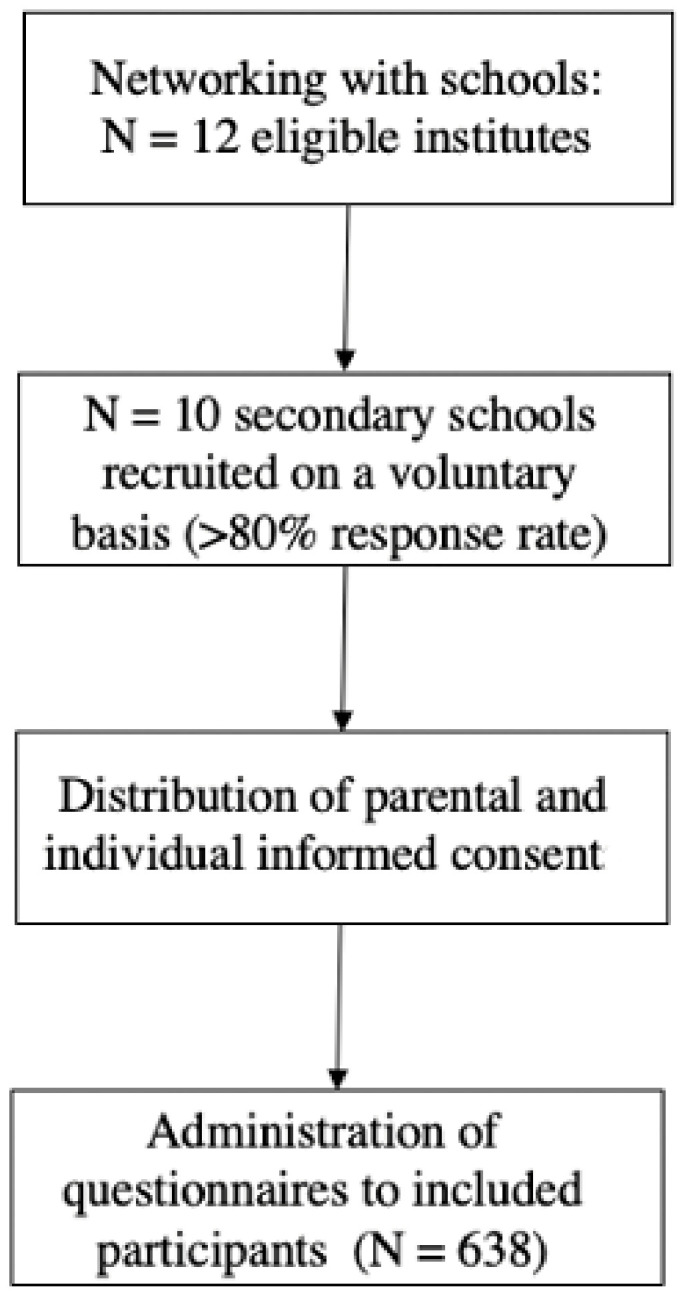
Flowchart of the recruitment process.

In total, 638 participants (53.9% boys; M_age_ = 15.65; SD = 1.32) were recruited on a voluntary basis from 10 secondary schools (grades 9–11) in the urban area of Rome and its surroundings. Data were collected during the assessment phase of an intervention project designed to reduce weight-based stigma and victimization. Schools were contacted through convenience sampling using networks from the authors’ institutions. After a detailed explanation of the study, parental and individual informed consent was acquired in each class two weeks before data collection (Figure 1). Students were invited to participate in the study without any restrictions. All protocols and procedures were approved by the Department of Psychology’s Institutional Review Board (prot. number 0001069).

### 2.2. Instruments

- Demographic information: respondents were asked to indicate their sex, age, class level, height (m), and weight (kg). BMI was computed using the standardized formula [body mass (kg)/height (m^2^)].
- Bullying victimization: the modified version of the “revised Olweus Bully/Victim Questionnaire” [50] adapted by Bacchini et al. [51] and widely used in Italy [52,53] was used. The questionnaire assessed 11 types of bullying, including direct (e.g., verbal offenses and physical aggression) and indirect forms (e.g., spreading rumors and exclusion from other group activities). Participants answered the questions referring to the previous six months. The questionnaire was completed after receiving a briefing from research authors on the standard definition of bullying, as previously indicated [52]. Responses were rated on a 5-point scale (1 = never; 2 = once/twice; 3 = 2/3 times a month; 4 = about once a week; 5 = several times a week). A total score of bullying victimization was computed by summing the scores of all items, with higher scores indicating a greater frequency of engaging in bullying victimization. The scale showed good internal consistency in the present study (ω = 0.848) as in previous results [51].
- CER strategies: the Italian short version [54] of the Cognitive Emotion Regulation Questionnaire (CERQ-18) [55] evaluates nine CER strategies: acceptance (e.g., I think that I have to accept the situation); putting into perspective (e.g., I tell myself that there are worse things in life); positive refocusing (e.g., I think of pleasant things that have nothing to do with it); positive reappraisal (e.g., I think I can learn something from the situation); positive refocusing (e.g., I think of pleasant things that have nothing to do with it); refocus on planning (e.g., I think about how to change the situation); rumination (e.g., I often think about how I feel about what I have experienced); catastrophizing (e.g., I continually think how horrible the situation has been); self-blame (e.g., I feel that I am the one who is responsible for what has happened); and other-blame (e.g., I feel that basically the cause lies with others). Responses were rated on a 5-point Likert scale, ranging from 1 (rarely) to 5 (almost always), with higher scores indicating a higher frequency of use of a certain cognitive CER strategy. In the present study, scores of the nine subscales were summed and categorized into dysfunctional and functional strategies, as indicated elsewhere [56]. As previously indicated [56], the composite scores of functional CER (ω = 0.907) and dysfunctional CER strategies (ω = 0.883) demonstrated good internal consistency.
- Psychological distress: the Italian version [57] of the Depression Anxiety Stress Scales (DASS-21) [58] consists of 21 items evaluating three facets of negative emotional states. Participants indicated how often they have reported symptoms in the previous week and responses were given on a 5-point Likert scale ranging from “always” (0) to “never” (4). These three dimensions have shown appropriate psychometric characteristics [58]. In the present sample, each subscale showed good reliability (Depression: ω = 0.888; Anxiety: ω = 0.877; Stress: ω = 0.875), as in the validation Italian study [57].

### 2.3. Data Analytic Strategy

Data were analyzed using Jamovi 2.3 [59] and Mplus 8.6 [60]. Preliminarily, descriptive statistics and zero-order correlations among the main variables under investigation were calculated. Subsequently, the indirect relationships between bullying victimization experienced in the previous six months and psychopathological symptoms (i.e., stress, anxiety, and depression) suffered in the previous week via general functional and dysfunctional CER strategies were tested within the structural equation modeling framework (SEM). To control for measurement error and the issue of attenuation in mediation analyses [61], all the constructs mentioned above were specified as single-indicator latent variables by estimating the error variances from their reliability. In line with Bollen [62], the error variances of the indicators were fixed at (1 − rxx) × s2, where rxx is the scale reliability and s2 is the sample variance. To partial out their effects, we included gender (0 = males, 1 = females), age, and BMI as covariates in the SEM using the full partial control approach [63]. The significance of the indirect effects was formally tested through bias-corrected bootstrap confidence intervals (5000 resamplings) [64]. After calculating critical values for the upper and lower 95% confidence limits, those with confidence intervals not encompassing zero were considered statistically significant. The bias-corrected bootstrap offers excellent performance in terms of statistical power, the accuracy of confidence intervals, and the overall control of Type I errors, especially when dealing with complex models involving multiple mediators [65]. Finally, we employed maximum likelihood with standard errors robust to non-normality as the parameter estimation method (MLR) [60] due to non-negligible deviations from the univariate normal distributions of the observed indicators (i.e., skewness and kurtosis > |1|) [66].

## 3. Results

### 3.1. Description of the Sample

In total, 194 students reported that they had never been bullied, whereas 444 students declared they had experienced at least one type of bullying victimization in the previous six months. The results are displayed in Table 1. The mean BMI was within the normal range (M = 21.68; SD = 4.09).

### 3.2. Bivariate Correlations

Descriptive statistics and bivariate correlations for the main constructs under investigation are reported in Table 2. All variables were approximately normally distributed, except for bullying victimization (skewness and kurtosis > |1|). To compensate for departures from univariate normality, MLR estimation was employed for further SEM analyses [63]. Bullying victimization was positively correlated with dysfunctional CER strategies (r = 0.297, *p* < 0.001), depression (r = 0.376, *p* < 0.001), anxiety (r = 0.338, *p* < 0.001), and stress (r = 0.329, *p* < 0.001). Dysfunctional CER strategies correlated with functional CER strategies (r = 0.419, *p* < 0.001), depression (r = 0.550, *p* < 0.001), anxiety (r = 0.523, *p* < 0.001), and stress (r = 0.606, *p* <.001). Lastly, functional CER strategies were significantly associated with depression (r = 0.111, *p* = 0.005), anxiety (r = 0.124, *p* = 0.002), and stress (r = 0.232, *p* < 0.001).

### 3.3. Mediation Model

**Figure 2:**
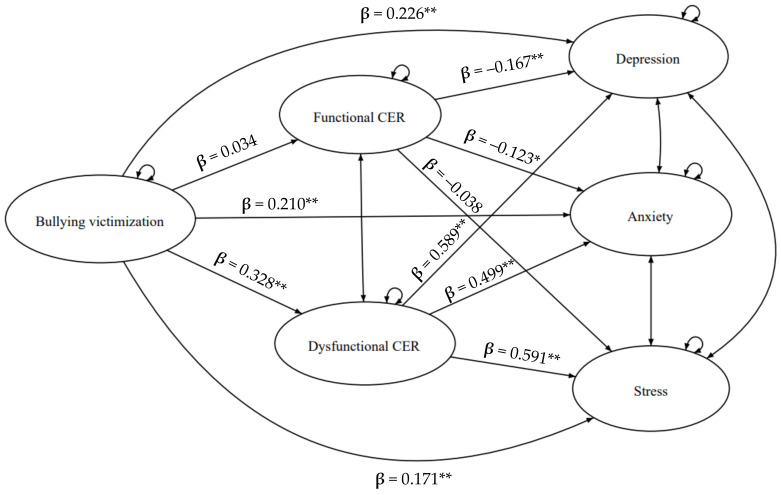
The proposed SEM. Note: Bullying victimization, CER strategies, depression, anxiety, and stress were posited as single-indicator latent variables. Covariates are not presented for the sake of clarity (i.e., gender, age, and BMI). Abbreviations: CER, cognitive emotion regulation. Standardized effects are displayed. * *p* < 0.01; ** *p* < 0.001.

The mediation model reported in Figure 2 was examined within the SEM framework. Notably, since the model had just been identified (i.e., 0 degrees of freedom), its fit was perfect by definition and could not be tested [67]. Overall, the model explained a substantial proportion of the variance in dysfunctional CER strategies (22%), depression (47%), anxiety (46%), and stress (52%), but not in functional CER strategies (2%).

More specifically, bullying victimization was positively related to dysfunctional CER (β = 0.328, *p* < 0.001). In turn, dysfunctional CER was significantly associated with depression (β = 0.589, *p* < 0.001), anxiety (β = 0.499, *p* < 0.001), and stress (β = 0.591, *p* < 0.001). The indirect effects supported our hypotheses (Table 3), highlighting the role of dysfunctional CER in mediating the impact of bullying victimization on depression (β = 0.193, 95% BCI 0.143–0.249), anxiety (β = 0.164, 95% BCI 0.119–0.215), and stress (β = 0.194, 95% BCI 0.144–0.246). Bullying victimization also affected depression (β = 0.226, *p* < 0.001), anxiety (β = 0.210, *p* < 0.001), and stress (β = 0.171, *p* < 0.001) directly; therefore, the SEM suggested the presence of partial mediation.

In contrast, bullying victimization did not contribute to functional CER (β = 0.034, *p* = 0.422). In turn, functional CER exerted a unique effect on depression (β = −0.167, *p* < 0.001) and anxiety (β = −0.123, *p* = 0.009). None of the indirect effects determined via functional CER were statistically significant (*ps* > 0.05; Table 3).

Concerning the covariates, females scored higher on dysfunctional CER (unstandardized B = 4.141, *p* < 0.001), functional CER (unstandardized B = 1.666, *p* = 0.008), depression (unstandardized B = 0.124, *p* = 0.020), anxiety (unstandardized B = 0.331, *p* < 0.001), and stress (unstandardized B = 0.227, *p* < 0.001). Moreover, BMI was positively associated with bullying victimization (β = 0.134, *p* = 0.006).

## 4. Discussion

The present study aimed to expand previous research on the association between bullying victimization and psychopathological symptoms in adolescents by evaluating the mediating role of functional and dysfunctional CER strategies. This study contributes to the literature on the role of emotion regulation processes in the implications of bullying victimization on adolescent mental health. The findings suggest that the relationships between being bullied by peers and mental difficulties may be both direct and indirect with the mediation of dysfunctional CER strategies.

Specifically, the first finding is consistent with previous studies evidencing a positive association between bullying victimization and each of the three dimensions of the DASS, supporting the well-known negative emotional consequences for bullying victimization and depression, anxiety, and psychological stress in adolescents [12,28,68,69]. Considering the cross-sectional nature of this study, the opposite path could also be reasonable. For example, research has demonstrated that adolescents who experience mental distress are particularly vulnerable to different forms of maltreatment and abuse [70]. Moreover, this bidirectional relationship could perpetuate bullying victimization through a vicious cycle of emotional maltreatment when students who are bullied and experience psychopathological distress may feel helpless, and thus may become more susceptible to acts of aggression [71]; psychopathological symptoms could inhibit their ability to cope with bullying [72]. For example, because depression is characterized by intense isolation, sadness, extreme pessimism, and loss of interest in previous pleasure activities, students experiencing bullying victimization may feel hopelessness and be incapable of objecting to abuse from their peers [68]. Moreover, previous research has suggested that the presence of anxiety and stress in victims can perpetuate the risk of being bullied [73]. Further longitudinal investigations are needed to estimate the direction of the link between bullying victimization and mental distress, as well as their mutual influence over time.

Returning to our mediational model, the second finding is that dysfunctional CER strategies are significantly associated with depression, anxiety, and stress. This evidence is also consistent with previous research and emphasizes the detrimental nature of maladaptive cognitive processes to regulate emotions in adolescence [43,44], as well as with the transdiagnostic role of dysfunctional CER, such as rumination and repetitive negative thinking, in contributing to psychopathology [44]. It has been observed that internal dysfunctional emotion regulation is strongly accompanied by psychopathological symptoms in youth [74], and adolescents with depression, anxiety, and stress symptoms report more problematic emotion regulation [75,76]. Longitudinal evidence has revealed that this link reflects bidirectional relationships [77], because psychopathological symptoms may also inhibit the individual’s ability of appropriately regulating emotions in response to negative stimuli [74,78]. For example, the inability to effectively manage or regulate emotional responses to daily events can lead to stress, depression, or anxiety in youngsters [79,80,81], and vice versa [82,83]. Further studies are needed on the use of experimental methods to assess the causal direction of these paths.

The present results indicate a non-significant association between bullying victimization and functional CER strategies, consistent with findings evidencing no direct relationship between these two constructs [84]. This result appears to indicate that the experience of being bullied is not associated with limited access to functional CER strategies from adolescents.

In contrast, the path from functional CER strategies to depression and anxiety was significant, substantiating previous studies in the literature [35,85]. However, as compared with dysfunctional CER strategies, weaker associations with psychological difficulties were observed, as previously reported [44,86]. A plausible explanation of the weaker associations found between functional CER and psychopathological symptoms could be that they are context-dependent, and can only be adaptive in certain circumstances (e.g., when the stressful event can be reformulated) [85].

Concerning the primary objective of this study, a significant indirect effect of dysfunctional CER strategies was found in partially explaining the link between bullying victimization and depression, anxiety, and stress. It is possible that disruptions in emotion regulation may lead to the modification of response to a stressor (e.g., bullying victimization, in our study), which, in turn, can impact individual mental health [87]. In this perspective, emotional responses associated with bullying victimization adversely affect adolescents’ cognitive regulatory system [88], and may thus result in psychological difficulties [40].

It has consistently been asserted that difficulties in emotion regulation contribute to the maintenance of emotional problems in youth [45,88], and have been regarded as transdiagnostic underlying mechanisms in the development of psychopathological symptoms from mid to late adolescence (e.g., depression) [89]. Some authors have suggested that less general use and a greater focus on specific functional and dysfunctional CER strategies strengthen the negative and positive correlation between being bullied and psychological distress [39,90], demonstrating that CER can also moderate this association. These findings encouraged future prospective studies to determine mechanisms (e.g., mediation) and conditions (e.g., moderation) related to CER through which adolescent mental functioning can be affected by bullying victimization. This topic is especially relevant because the role of functional CER strategies was not significant in the mediation analysis of the present study. Notwithstanding previous research which found that functional emotion regulation processes are important for reducing the negative effects of peer victimization on mental health difficulties in youth [46], this finding was not supported by our results. It seemed that the significant association between bullying victimization and psychopathology was not due to the adolescents’ diminished engagement in functional CER strategies. It is possible that the functional CER strategies were not meaningful enough to predict a decrease in symptoms associated with being bullied. A reasonable explanation of this finding may be that the components of functional emotion regulation processes may not be sufficiently structured in adolescence [91], and thus, are less refined to respond to stressful events such as bullying victimization. Indeed, it is well known that emotion regulation strategies are more effective as protective factors against psychological difficulties with growing age [92]. Future longitudinal research should focus on evaluating the association between adolescents’ bullying victimization and functional CER in predicting the onset of psychopathology, considering context-dependent factors such as the individual competence in emotion regulation acquired. Moreover, considering that, in the present results, functional and dysfunctional CER were positively correlated, as previously indicated [56], it is possible that the adaptive strategies were not sufficiently developmentally established to exert opposite effects on adolescent mental adjustment. Further research is needed to address the developmental trajectory of the reciprocal associations between tendencies to use dysfunctional and functional CER strategies in handling bullying victimization.

### 4.1. Limitations

We are aware that our research has some limitations. First, its cross-sectional nature prevents drawing conclusions about causality/directions of influence. This point highlights the urgency of further longitudinal studies addressing the mediation role of CER in the relationship between bullying victimization and mental health symptoms in adolescents. More specifically, future research should examine whether bullying victimization could predict dysfunctional emotion regulation over time—as previously suggested by retrospective studies [93]—that, in turn, would be a risk factor for the development of psychopathological symptoms consistently with existing evidence [94]. Second, the mere use of self-reported measures could be affected by social desirability bias. Future studies should employ other more rigorous methods, such as experimental tools to assess processes associated with emotion regulation [95]. Additionally, qualitative methods may be useful, such as structured interviews or daily diaries, for the collection of subjective data on the experience of bullying victimization, emotion regulation, and psychopathology. Moreover, considering the association of bullying victimization with socioeconomic status and peer/parental support consistently found in the literature [15], these aspects should be assessed in future studies.

### 4.2. Conclusions

Despite these weaknesses, this study suggests that interventions focused on targeting dysfunctional cognitive processes to regulate the emotions of peer-victimized adolescents may alleviate the psychological maladjustment associated with this stressful experience [47]. For example, emotion coaching can be effective in helping adolescents to self-regulate their emotions at school, promoting emotional competencies and positive peer interactions [96,97]. School prevention and treatment programs that can encourage adolescents to modify maladaptive patterns of CER that are typically used to cope with experienced bullying situations are illustrated in the literature [98]. For instance, metacognitive therapy (MCT) can be a valid short-term intervention to reduce adolescent dysfunctional CER and associated maladaptive outcomes [99]. Another example is emotion regulation training (ERT), which can be effective in increasing positive emotions and promoting personal strengths and resiliency in students experiencing bullying victimization [100]. Notably, since CER strategies begin to develop during the first years of life [101], it is essential to promote such interventions in the preschool years.

To conclude, our results suggest that maladaptive forms of CER strategies might be underlying mechanisms in the link between bullying victimization and emotional difficulties in adolescence. This is a particularly meaningful contribution because these problems often exhibit their first onset in adolescence, suggesting the urge to plan preventive and treatment interventions focused on experiences of victimization and their consequence on mental health in this life period. Nevertheless, these findings support the importance of contrasting bullying episodes and involvement, especially in the school context, in order to limit its negative effects on the psychological adjustments of adolescents.

## Figures and Tables

**Table 1:** Frequency rates for each of the bullying victimization types (n = 638).

Bullying Victimization	0	1	2	3	4
Teasing for physical appearance	486	67	68	9	8
Teasing for other reasons	442	54	97	25	20
Name-calling	467	87	52	17	15
Physical bullying	549	55	23	3	8
Threatens	565	40	24	3	6
Spreading rumors	486	73	51	14	14
Ignoring others	496	75	42	13	12
Stealing	478	109	39	7	5
Exclusion from sports activities	558	41	23	8	8
Exclusion from group activities	518	65	40	5	10
Exclusion from parties	490	71	60	3	14

0 = never; 1 = once/twice; 2 = 2/3 times a month; 3 = about once a week; 4 = several times a week.

**Table 2:** Descriptive statistics and bivariate correlations for the main variables under investigation.

Variable	Mean (SD)	Skewness	Kurtosis	1	2	3	4	5
1. Bullying victimization	16.22 (5.79)	2.36	6.82					
2. Dysfunctional CER strategies	21.92 (6.76)	−0.15	−0.44	0.297 **				
3. Functional CER strategies	31.09 (8.25)	−0.57	0.21	0.025	0.419 **			
4. Depression	1.03 (0.75)	0.57	−0.38	0.376 **	0.550 **	0.111 *		
5. Anxiety	0.97 (0.73)	0.72	−0.12	0.338 **	0.523 **	0.124 *	0.745 **	
6. Stress	1.29 (0.72)	0.24	−0.52	0.329 **	0.606 **	0.232 **	0.761 **	0.801 **

Abbreviations: CER, cognitive emotion regulation; SD, standard deviation. * *p* < 0.01; ** *p* < 0.001.

**Table 3:** Estimates of the indirect effects along with bootstrap-based confidence intervals.

Indirect Effect	Standardized β	95% BCI
Bullying–Dysfunctional CER—Depression	0.193	0.143 to 0.249
Bullying–Dysfunctional CER—Anxiety	0.164	0.119 to 0.215
Bullying–Dysfunctional CER—Stress	0.194	0.144 to 0.246
Bullying–Functional CER—Depression	−0.006	−0.023 to 0.007
Bullying–Functional CER—Anxiety	−0.004	−0.020 to 0.005
Bullying–Functional CER—Stress	−0.001	−0.011 to 0.002

Abbreviations: BCI, bias-corrected bootstrap-based confidence interval; CER, cognitive emotion regulation.

## Data Availability

The data presented in this study are available on request from the corresponding author. The data are not publicly available due to privacy and ethical restrictions.
